# Pregnancy and working conditions in the hospital sector: a scoping review

**DOI:** 10.47626/1679-4435-2023-947

**Published:** 2023-04-18

**Authors:** Soraya Wingester Vasconcelos, Joana C Guedes, Elizabeth Costa Dias, Alexandra Matias

**Affiliations:** 1 Programa Doutoral em Segurança e Saúde Ocupacionais, Departamento de Minas, Faculdade de Engenharia, Universidade do Porto, Porto, Província Douro Litoral, Portugal; 2 Laboratório Associado de Energia, Transportes e Aeronáutica, Faculdade de Engenharia, Universidade do Porto, Porto, Província Douro Litoral, Portugal; 3 Departamento de Medicina Preventiva e Social, Faculdade de Medicina, Universidade Federal Minas Gerais, Belo Horizonte, MG, Brazil; 4 Departamento de Ginecologia e Obstetrícia, Hospital de S. João, Faculdade de Medicina, Universidade do Porto, Porto, Província Douro Litoral, Portugal

**Keywords:** absenteeism, health personnel, pregnancy, sick leave, hospital units, absenteísmo, pessoal de saúde, gravidez, licença médica, unidades hospitalares

## Abstract

The different areas and work environments in the hospital sector have a complex set of occupational risk factors that can negatively impact the health of pregnant workers. Illness among this workforce results in sick leave due to work-related diseases and pregnancy, with high absenteeism. The main objective of this study was to review the available literature on the gestational and occupational risks to which pregnant health workers are exposed, causes of absenteeism, and issues related to maternity protection and work in the hospital sector. The authors used online databases to identify papers published in English from 2015 to 2020, based on the PRISMA Extension for Scoping Reviews and three steps of Snowballing. The study reviewed 18 peer-reviewed scientific articles that address pregnancy, work, absenteeism, and maternity protection. Most studies used a quantitative approach (12) and cohort studies in particular (6). The distribution of articles by themes was as follows: pregnancy, health and safety at work (11); pregnancy, health conditions, and absenteeism (13); and work and maternity protection (10). Some inferences were possible from the themes raised. However, the results revealed a gap and the need for specific studies for healthcare workers in the hospital sector, focusing on maternity. This review contributes to more in-depth studies on developing programs, actions, and legislation to protect maternity in hospital working environments.

## Abstract (pt)

As diferentes áreas e ambientes de trabalho do setor hospitalar apresentam uma complexidade de fatores de risco que podem impactar negativamente a saúde das trabalhadoras grávidas. O adoecimento dessa força de trabalho resulta em afastamentos por doenças relacionadas ao trabalho e à gravidez, com alto absenteísmo. O objetivo principal deste estudo foi revisar a literatura disponível sobre os riscos gestacionais e ocupacionais a que estão expostas as trabalhadoras de saúde grávidas, causas de absenteísmo e questões relacionadas à proteção da maternidade e do trabalho no setor hospitalar. Bases de dados online foram usadas para identificar artigos em inglês publicados de 2015 a 2020, com base no PRISMA Extension for Scoping Reviews e três etapas de *snowballing*. Este estudo incluiu 18 artigos científicos revisados por pares que abordam questões relativas à gravidez, absenteísmo e proteção à maternidade no trabalho. A maioria dos estudos utilizou abordagem quantitativa (12), com ênfase nos estudos de coorte (6). Distribuição dos artigos por temas: gravidez, saúde e segurança no trabalho (11); gravidez, intercorrências e absenteísmo (13); e proteção ao trabalho e maternidade (10). Algumas inferências foram possíveis a partir dos temas levantados. No entanto, os resultados evidenciaram a lacuna e a necessidade de estudos específicos para trabalhadores de saúde do setor hospitalar, com enfoque na maternidade. Esta revisão pode contribuir para estudos mais aprofundados sobre o desenvolvimento de programas, ações e legislações de proteção à maternidade no ambiente de trabalho do setor hospitalar.

## INTRODUCTION

Working in hospitals involves a complex set of occupational risk factors for workers’ health. Due to its nature and the context of the health system, a hospital may be the setting that brings together the most significant number of workers from different categories, work situations, and occupational exposures. As a result, it provokes several physical and psychological illnesses, with consequent absenteeism that is considerably higher when compared to other professional categories and productive sectors.^1^

Considering all workers who produce health-related products and provide services, the workforce in health-related economies was estimated at 234 million worldwide in 2013.^2^ Of these, 71 million were workers in health occupations. The proportion of women working in the health and social sector was almost a third higher than the proportion of women in overall employment. Globally, more than 70% of workers in this sector were women, which highlights the significant majority of women in the health sector and its importance as a source of employment.^2,3^

Several agents present in the hospital work environment can pose risks to workers’ health due to the typical characteristics of occupational exposure, which is primarily repetitive and prolonged. Biological, chemical, physical, biomechanical, and psychosocial risk factors can negatively impact the health of female workers who are pregnant, nursing, or of childbearing age.^2,4,5^

Risk factors such as inhalational anesthetics, ionizing radiation, antineoplastic agents, and viruses pose risks to sexual and reproductive health, including increases in congenital anomalies. Other important factors are lifting and carrying loads, inappropriate postures, and psychosocial risk factors involving shift work, irregular hours, and stress related to the way work is organized.^5^

Furthermore, chronic diseases such as diabetes and gestational hypertensive syndromes (gestational hypertension, pre-eclampsia, eclampsia), or hemorrhagic disorders (abortion, placenta previa, and placental abruption),^6,7^ can be triggered or aggravated by working conditions.

According to the International Labor Organization (ILO), absenteeism is paid or unpaid absence of a worker for more than 1 working day, by medical order or otherwise, when he/she was expected to be present.^8^ Short-term absences are usually related to organizational and stress factors at work, such as personal restrictions, organizational tension, career limitations, and work overload. The frequency of short-term absenteeism can be an indicator of the organizational climate. On the other hand, long-term absenteeism is more directly related to disease itself and may indicate a worker’s health condition.^1^

A descriptive cross-sectional study at the Mazandaran University of Medical Sciences in Iran evaluated employees’ absenteeism (sick leave) in 2010. The average number of sick days was 2 ± 1, and about 60% of employees were women. The most common causes of absenteeism were respiratory diseases (colds and flu - 212), neck and back pain (118), fever and headache (71), and infectious diseases (diarrhea and vomiting - 88).^9^

A number of studies in Sweden and Norway were included in a systematic review to assess the effectiveness of interventions in healthcare settings or workplaces targeting sickness absence among pregnant women. The frequency of women on sick leave from work was lower in the intervention groups and significantly lower among pregnant women who participated in a 12-week physical exercise program.^10^

The answers to certain questions are essential to protection of maternity in the hospital sector: “What conditions cause absenteeism among pregnant workers in the hospital sector?”, “What are the possible associated occupational factors and measures to protect maternity?”, and “How does maternity interfere with professional life?”

Therefore, the main objective of this scoping review was, to consult the existing literature to compile and describe the gestational and occupational risks to which pregnant workers are exposed, the causes of absenteeism, and issues related to maternity protection and work in the hospital sector.

## METHODS

In this review, the Preferred Reporting Items for Systematic Reviews and Meta-Analyzes - PRISMA Extension for Scoping Reviews (PRISMA-ScR) - was used^11^ in the search process and selection of studies and for presentation and discussion of results. Scoping review methods are helpful when topics are complex or have heterogeneous literature and it is necessary to extract the data to map it. Also, scoping reviews can summarize a field, identify gaps for future research, and guide projects.^12^

### ELIGIBILITY CRITERIA

Articles were selected using the following eligibility criteria: English language, publication period January 2015 to December 2020, observational studies, qualitative studies, intervention studies, quality improvement studies, and peer-reviewed studies. These study types were included as criteria to ensure a comprehensive synthesis of all terms and themes related to the review. The study population should consist of healthcare professionals of childbearing age or pregnant, including those undergoing training in the area (e.g., residents), from the various worker categories and hospital sectors. Reported data should include working conditions, the pregnant worker in the hospital sector, common complications during pregnancy, absenteeism, and social benefits.

Papers were excluded if they did not fit into the conceptual framework of the study based on the Population, Concept, and Context (PCC) strategy for scoping review, where P = pregnant workers; C = work, pregnancy, maternity protection; and C = hospital sector. Additionally, papers about animal trials, non-occupational practices, and patient care and those reporting maternity leave as the only outcome were excluded.

When the results of the articles included participants different from the target population of this review, we sought to use stratified data identifying pregnant workers, when reported, and aspects of occupational hazards and working conditions also common to pregnant, non-pregnant, or childbearing women.

### INFORMATION SOURCES

The following bibliographic databases were searched to identify potentially relevant studies: Scopus, PubMed, and Web of Science. All citations identified were imported into the JabRef 5.1 bibliographic reference manager, via BibTeX files generated by the three databases. Duplicates were removed, first with the aid of JabRef and then manually by date.

### SEARCH

A preliminary search strategy was constructed in the Scopus database, using words related to the study subject to help define the keywords and their combinations. The other databases were then consulted using the same process. The memory of the SCOPUS search strategy is presented in Chart 1, and the eligibility assessment steps, the Snowball Technique, and the search in specialized journals are shown in Table 1. Boolean operators, and truncation, in work* and pregnan*, were used in the search.

**Chart 1:** Search strategy^*^ for potentially qualifying articles in SCOPUS- Search period: October 31, 2020 to November 17, 2020

KWC		Search of citations
1 KWC	work^*^ And pregnan^*^ And hospital	TITLE (work^*^) AND TITLE-ABS-KEY (pregnan^*^) AND TITLE-ABS-KEY (hospital)) AND (LIMIT-TO (PUBYEAR, 2020) OR LIMIT-TO (PUBYEAR, 2019) OR LIMIT-TO (PUBYEAR, 2018) OR LIMIT-TO (PUBYEAR, 2017) OR LIMIT-TO (PUBYEAR, 2016) OR LIMIT-TO (PUBYEAR, 2015)) AND (LIMIT-TO (DOCTYPE, “ar”)) AND (LIMIT-TO (SRCTYPE, “j”)) AND (LIMIT-TO (LANGUAGE, “English”))
2 KWC	absenteeism OR sick leave AND hospital care personnel AND pregnancy	ALL (absenteeism) OR ALL (sick AND leave) AND ALL (hospital AND care AND personnel) AND ALL (pregnancy)) AND (LIMIT-TO (PUBYEAR , 2020) OR LIMIT-TO (PUBYEAR , 2019) OR LIMIT-TO (PUBYEAR , 2018) OR LIMIT-TO (PUBYEAR , 2016) OR LIMIT-TO (PUBYEAR , 2015)) AND (LIMIT-TO (DOCTYPE , “ar”)) AND (LIMIT-TO (SRCTYPE, “j”)) AND (LIMIT-TO (LANGUAGE , “English”))
3 KWC	work^*^ And pregnan^*^ And sick absence And hospital	TITLE-ABS-KEY (work^*^ AND pregnan^*^ AND sick AND absence AND hospital) AND (LIMIT-TO (PUBYEAR, 2019) OR LIMIT-TO PUBYEAR, 2018)) AND (LIMIT-TO (DOCTYPE, “ar)) AND (LIMIT-TO (SRCTYPE, “j”)) AND (LIMIT-TO (LANGUAGE, “English”))

* The strategy used in the Scopus database was applied to PubMed and Web of Science.

Screening criteria: period (2015-2020), type of document (article), type of source (journal), language (English). Boolean operators (AND, OR) and truncation in the terms pregnan^*^ and work^*^. Search within: Article title - Abstract - Keywords (TITLE-ABS-KEY).

KWC = keyword combinations

**Table 1:** The steps for importing citations into JabRef, selection, eligibility, and the total number of articles - Period: November 16, 2020 to January 5, 2021

Step		Total
Importing data into JabRef without duplicates	From SCOPUS, PubMed, and Web of Science, after identifying the records. New entries only with manual removal of duplicates.	1,051
First selection of articles from SCOPUS, PubMed, and Web of Science by title and abstract, on November 16, 2020	1st step - Search criteria - keywords (title= and abstract=) in the JabRef search engine: title=absenteeism and abstract=hospital; title=absenteeism and abstract=pregnancy; title=pregnancy and abstract=hospital; title=absence and abstract=hospital; title=sick and abstract=pregnancy; title=hospital and abstract=sick; title=sick and abstract=absence; title=work and abstract=pregnancy. Note: until exhaustion.	13
	2nd step - inclusion and exclusion - manual by title and abstract	
	Inclusion criteria: target population/participants - pregnant women workers; concept/idea - absenteeism of female workers during pregnancy; context: hospital sector; types of studies - all except trial.	
	Exclusion criteria: type of outcome (maternity leave and presenteeism); non-worker; the population beyond childbearing age; working in a sector other than healthcare; animal and laboratory/testing studies.	
Second selection of articles from SCOPUS, PubMed, and Web of Science by title and abstract, on November 17, 2020	Manual selection from the first selection, observing the same inclusion and exclusion criteria	7
Third selection of articles - First application of Snowball technique to the references of articles selected from the databases, November 10, 2020 to November 12, 2020	Exclusion criteria: year of publication (outside the period 2015-2020). Inclusion criteria: the same terms as in the previous queries.	0
Fourth selection of articles - Second application of Snowball technique to the articles of two authors with most articles found in the databases and related articles, November 23, 2020 to November 24, 2020	Exclusion criteria: year of publication (outside the period 2015-2020). Inclusion criteria: the same terms as in the previous queries.	0
Fifth selection of articles - search in specialized magazines, November 28, 2020 to January 5, 2021	Keywords used for the search: sickness absence, pregnant, hospital, sick leave, occupational health, healthcare worker, and occupational.	10
Sixth selection of articles - Third application of Snowball technique to the references of selected articles in the specialized magazines, January 5, /2021	Exclusion criteria: the year of publication (outside the period 2015-2020). Inclusion criteria: the same terms as in the previous queries.	1
Inclusion of all additional citations identified through other sources	Specialized magazines and third Snowball stage	11
Inclusion of full-text articles to be assessed for eligibility after records screening	1,051 (JabRef) + 10 (magazines) + 1 (third Snowball stage)	1,062
Exclusion of ineligible articles	After second selection by title and abstract	1,044
Inclusion of articles selected for the review		18

Additional articles were identified by manual searches of the reference lists of all included papers, comprising the first Snowball stage. An additional search was carried with most articles initially selected from the databases, comprising the second Snowball stage.

Specialized magazines on occupational health and safety (OHS), occupational medicine, and the journals of professional health categories were searched to increase the number of articles using the following search terms in the title, abstract, or main text: sickness absence, pregnant, healthcare worker, hospital, sick leave, occupational health, sickness absence, occupational. A third snowball stage searched the references of the articles selected from the specialized magazines.

### DATA CHARTING PROCESS

In accordance with the review’s objective, a data charting form was independently developed to determine which variables to extract from the included papers. It was continuously updated during reading of abstracts and articles according to similarities and common themes found in the articles. This tool was used to capture relevant information on key study characteristics and detailed information on pregnancy and maternity, health conditions, health and safety at work, and absenteeism.

### DATA ITEMS

Data were extracted on article characteristics (e.g., authors, year, title, country of origin, and journal) and study characteristics (e.g., type of study, research subject, research objectives/questions, methodology, and main findings). The themes defined for discussion of the results were: pregnancy, health and safety at work; pregnancy, health conditions, and absenteeism; and work and maternity protection.

### SYNTHESIS OF RESULTS

The collected data were organized to present the range of evidence identified that meet the objectives of the scoping review. A flow diagram illustrating the article search and selection process was prepared according to PRISMA-ScR. The data from the data charting form were summarized in tables by author, title, type of study, the country in which the study was conducted, and publishing journal, and by research subjects and main findings.

## RESULTS

### CHARACTERISTICS OF ARTICLES

**Figure 1:**
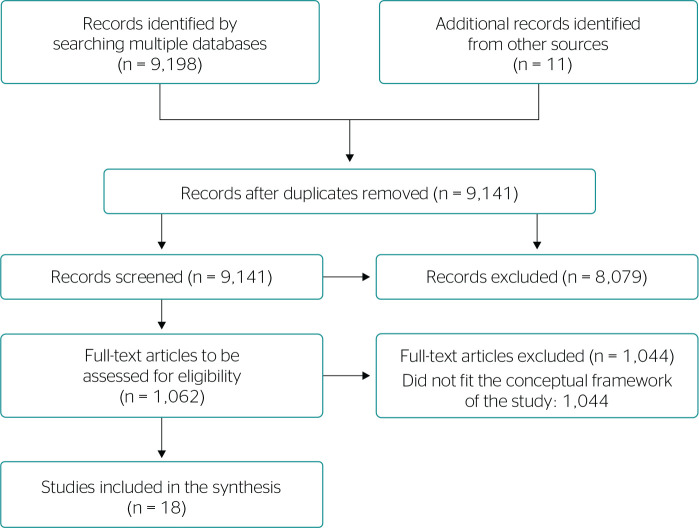
Flow diagram illustrating the article search and selection process for the scoping review.

Eighteen articles were included after searches in databases and specialized journals and three steps of Snowballing, as shown in Figure 1. Most articles were published in 2019 (7) and 2017 (5). Countries that stood out were Denmark (6), Brazil (2), and Spain (2).

The journals from which the highest number of articles were selected were *Occupational and Environmental Medi*cine and the *Scandinavian Journal of Work, Environment & Health* (3 each), followed by the *International Archives of Occupational and Environmental Health* (2). Most studies used a quantitative approach (12), with emphasis on cohort studies (6) among the observational, analytical studies (7) and on cross-sectional studies (4) among observational, descriptive studies (5) (Table 2).

**Table 2:** Studies included in the scoping review by author, title, type of study, country in which the study was conducted, and journal of publication

Author(s)	Title	Type of study	Country	Journal
Hansen et al.^13^	Occupational exposures and sick leave during pregnancy: results from a Danish cohort study	Analytical, observational	Denmark	Scandinavian Journal of Work, Environment & Health
Soteriades^14^	A workplace modified duty program for employees in an oncology center	Simple descriptive analysis	Republic of Cyprus	Work
Park et al.^15^	Adverse pregnancy outcomes in healthcare workers: a Korean nationwide population-based study	Descriptive, observational	Korea	International Archives of Occupational and Environmental Health
Gravel et al.^16^	Protecting pregnant workers while fighting sexism: work - pregnancy balance and pregnant nurses’ resistance in Quebec hospital	Qualitative	Canada	New Solutions
Backhausen et al.^17^	The prevalence of sick leave: Reasons and associated predictors - A survey among employed pregnant women	Descriptive, observational	Denmark	Sexual & Reproductive Healthcare
Truong et al.^18^	Sick leave and medication use in pregnancy: a European web-based study	Descriptive, observational	Norway (12 European countries)	BMJ Open
Gottenborg et al.^19^	You can’t have it all: the experience of academic hospitalists during pregnancy, parental leave, and return to work	Qualitative	United States	Journal of Hospital Medicine
Hammer et al.^20^	Night work and hypertensive disorders of pregnancy: a national register-based cohort study	Analytical, observational	Denmark	Journal of Work, Environment & Health
Probst et al.^21^	Implementation, mechanisms and effects of maternity protection legislation: a realist narrative review of the literature	Realistic narrative review	Switzerland	Scandinavian Journal of Work, Environment & Health
Begtrup et al.^22^	Night work and miscarriage: a Danish nationwide register-based cohort study	Analytical, observational	Denmark	International Archives of Occupational and Environmental Health
Hammer et al.^23^	Night work and sick leave during pregnancy: a national register-based within-worker cohort study	Analytical, observational	Denmark	Occupational and Environmental Medicine
Villar et al.^24^	Occupational risk during pregnancy and sick leave in a cohort of workers from Parc de Salut Mar (Barcelona, Spain)	Analytical, observational	Spain	Gaceta Sanitaria
Ménage et al.^25^	How junior doctors live their work during pregnancy? A qualitative study in France	Qualitative	France	Gynécologie Obstétrique Fertilité & Sénologie
Villar et al.^26^	Working conditions and absence from work during pregnancy in a cohort of healthcare workers	Analytical, observational	Spain	Occupational and Environmental Medicine
Rocha et al.^27^	Sickness absenteeism among health care workers in a public hospital in São Paulo, Brazil	Descriptive, observational	Brazil	Revista Brasileira de Medicina do Trabalho
Hammer et al.^28^	Night work and postpartum depression: a national register-based cohort study	Analytical, observational	Denmark	Scandinavian Journal of Work, Environment & Health
Trettene et al. ^29^	Absenteeism and the Technical Safety Index of a tertiary hospital nursing team	Descriptive, observational	Brazil	Revista da Escola de Enfermagem da USP
Masood & Nisar^30^	Crushed between two stones: competing institutional logics in the implementation of maternity leave policies in Pakistan	Qualitative	Pakistan	Gender, Work and Organization

The subjects and main findings of the review are described in Table 3. Regarding the themes selected for discussion, “pregnancy, health and safety at work” was broached in 11 articles, “pregnancy, health conditions, and absenteeism” in 13, and “work and maternity protection” in 10. The sub-themes that appeared most often in the studies were: absences from work/absenteeism (5); pregnancy complications (5); legislation - maternity leave policies (4); medical leave (4); working conditions (3); and night work (4).

**Table 3:** Studies included in the scoping review by author, research subject, and main findings

Authors	Research subjects	Main findings
Hansen et al.^13^	Working conditions and processes; absence from work during pregnancy.	In this population-based cohort of employed women, non-sitting work postures, lifting, shift work, and high job strain were associated with increased risk for sick leave after 10-29 completed pregnancy weeks. Changes in the work environment for pregnant women may reduce sick leave.
Soteriades^14^	Modified working hours (tasks and workload) and partial and temporary incapacity for work	The prevalence of participation in the program was 3%. Only women used the program and the highest percentages of employees assigned to modified service were due to pregnancy (50%) or back pain (25%).
Park et al.^15^	Complications of pregnancy among health workers	Health professionals had a higher adjusted OR in almost all obstetric consequences. Miscarriage, abortion threat, preterm labor, and intrauterine growth retardation showed higher adjusted OR in the working group than in the non-working group.
Gravel et al.^16^	OHS legislation, the adaptation of the workplace for pregnant women, precautionary leave, and gender discrimination	In the profession and hospitals studied, the existence of preventive/precautionary leave and its place in the OHS legislation have allowed pregnant nurses to remain active, maintain their economic independence, and protect their health. Leave with presentation of a medical certificate decreased and workers were kept in the workplace for longer, adapting their work assignments to the state of pregnancy.
Backhausen et al.^17^	Sick leave and self-reported reasons for leave during pregnancy	The prevalence of sick leave was 56% of pregnant women employed in the first 32 weeks of pregnancy. More than one in four reported long-term sick leave (> 20 days, continuous or intermittent). Lower back pain was the reason most often mentioned. Less than one in ten said the sick leave was due to working conditions. Positive predictors of long-term sick leave were multiparity, lower back pain before pregnancy, and mental illness, while higher education was a negative predictor.
Truong et al.^18^	Policies, standards, and reasons for sick leave during pregnancy (focus on drug use), on a multinational level	Women who used medication were more likely to be on sick leave (acute illness). The various sick leave patterns across countries partially reflected differences in sick leave policies. Thus, sick leave in pregnancy is also affected by other national differences, and rates vary significantly across European countries.
Gottenborg et al.^19^	Challenges and solutions to support academic doctors working in hospitals; maternity leave and return to work as a teacher.	Participants reported the following challenges: lack of paid parental leave and the associated financial penalties, loss of career opportunities, physical challenges associated with pregnancy, decreased productivity, and the amount of time and effort involved in breastfeeding. They shared ideas for future solutions to alleviate the challenges posed to working medical mothers.
Hammer et al.^20^	HDP and the different dimensions of nightwork	Of the 18,724 workers, 60% worked at least one night shift in the first 20 weeks of pregnancy. Working consecutive night shifts and rapid returns after night shifts during the first 20 weeks of pregnancy were associated with an increased risk of HDP, mainly among obese women.
Probst et al.^21^	Implementation and effects of MPL	The implementation of MPL is deficient in most of the countries studied. Allowing pregnant women to leave work for preventive and medical leave is favored over workplace accommodations or relocation to other sectors and tasks. The delay between the conception and implementation of maternity protection is a significant barrier to its effectiveness at the individual, physical, social, and macrosocial levels. Lousy labor relations and discrimination can impede its implementation.
Begtrup et al.^22^	Night work and sick leave due to miscarriage	Increased risk of miscarriage among women who worked at night in the previous week and among women with cumulative numbers of night shifts. There was a 32% increase in risk after the 8th week of pregnancy for two or more night shifts in the previous week compared to women who did not work night shifts in the last week.
Hammer et al.^23^	Risk of getting sick the day after the night shift at work and sick leave during pregnancy	Among Danish public hospital workers, night shifts significantly longer than 12 hours during pregnancy increased the risk of getting sick the next day, regardless of personal factors and time-invariant confounders, in all trimesters of pregnancy.
Villar et al.^24^	Use of benefits: PRE and ITcc.	As the pregnancy progressed, the number of PRE (32%) and ITcc (68%) increased. In the end, most workers were absent. 50% of the pregnant women worked until day 187. Of the theoretical total number of working days in the cases (119,840 days; 280 days/pregnancy), two-thirds remained active at work, and, of the absent third, 68% were due to ITcc and the rest were due to PRE.
Ménage et al.^25^	Working conditions and absence from work during pregnancy	From the coding of the interviews, it was possible to distinguish two main themes: work and parenting. As for work, residents expressed doubts about their own work, interpersonal relationships, and adaptation to work during pregnancy and when returning to work.
Villar et al.^26^	Working conditions during pregnancy and the use of POR and sick leave benefits	Three pregnancy trajectories of workers were identified in the study: absences covered mainly by sick leave, absences covered by POR, and few absences. The POR benefit was used to cover absences of women highly exposed to occupational risk factors (ergonomic, safety, hygiene, and psychosocial). Sick leave was the benefit most used by pregnant workers and was not associated with exposure to occupational risk.
Rocha et al.^27^	Occupational absenteeism in the hospital sector	Most workers were female, with a relatively high average age and many years of service. The Occupational Safety and Medicine Service was sought mainly by nursing assistants, followed by nurses and doctors. The absenteeism rates due to illness proved adequate and pointed to a profile characterized by chronic diseases (prolonged absence), with the highest rates corresponding to emergency room workers. The main conditions associated with frequency and absences were musculoskeletal diseases and mental and behavioral disorders.
Hammer et al.^28^	Night work, pregnancy, and PPD	Most of the workers were nurses or doctors. No increased risk of severe PPD was observed for any of the dimensions of night work analyzed. There was an increased risk of PPD among women who stopped working night shifts after the first trimester of pregnancy. The results do not support night work during pregnancy as a risk factor for severe PPD among hospital workers.
Trettene et al.^29^	Occupational absenteeism in a tertiary hospital nursing team	The absenteeism rate of the nursing team was 21.5%, mainly due to maternity leave of nurses and medical leave of nursing technicians, while the Technical Safety Index was 40%.
Masood & Nisar^30^	Different logics involved in the implementation of MLP that prevail in various institutions: family, state, and profession	The design of MLP reflects the gender political ideology of Pakistani society: “ideal” women belong in the private sphere. Patriarchal logic rewards masculine qualities of absolute commitment to the medical profession. Some obstacles to accessing MLP are administrative processes, complicated policies, and meeting multiple eligibility requirements. The data suggest that women who work as regular employees in public hospitals have better access to maternity policies than those in the private sector due to a lack of social security, corporate strategies to escape maternity benefits, and a lack of options to extend leave. It is vital to recognize discrimination against pregnancy in addition to gender discrimination.

HDP = hypertensive disorders of pregnancy; ITcc = temporary disability due to common contingency; MLP = maternity leave policy; MPL **=** maternity protection legislation; OHS = occupational health and safety; OR = odds ratio; POR = occupational risk benefit in pregnancy; PPD = postpartum depression; PRE = risk benefit during pregnancy.

## DISCUSSION

One methodological challenge in analyzing the studies regarding the topics of interest was interpreting the data collected, considering the approach, design, and population studied. Comparing studies with different analysis methods can distort the results.

Dealing with organizational characteristics at the levels of health services and individuals can lead to flaws and fragility of methods, making it impossible to extrapolate findings.^31^ In addition, some studies lacked an approach that considers the workers’ perceptions and knowledge^32^ in the analysis or explanation of the studied phenomena.

### PREGNANCY, HEALTH AND SAFETY AT WORK

The findings related to work organization issues, which were analyzed in several articles,^13-17^ corroborate the evidence in the literature on the impacts of occupational risk factors on the health of pregnant workers^2,4,5^, as well as in the use of medical leave or “precautionary leave”, in the impossibility of readjusting the work environment or reassignment to other tasks. Work adjustment was associated with reduced sick leave during pregnancy in the Norwegian Mother and Child Cohort Study (MoBa).^33^

Risk factors such as non-sitting working postures, lifting weight, shift work, number of night shifts, hours worked, and high stress at work^5,13^ underscore the need for task modification programs.^14,34^ Programs for workers with partial and temporary incapacity to work have multiple benefits. They contribute to reduced exposure to risk factors and health impacts, such as back pain, hypertensive disorders in pregnancy,^20^ and severe postpartum depression (PPD),^28^ besides helping pregnant women to keep working, avoiding financial and professional losses.^35^

Retaining a skilled employee, increasing worker productivity, and eliminating the training costs of a new employee directly benefit employers.^35^ However, the literature points to employers’ resistance to making adaptations in the workplace, as shown in a study by Malenfant & De Koninck: most eligible pregnant workers were placed on leave, despite protective reassignment legislation.^36^

### PREGNANCY, HEALTH CONDITIONS, AND ABSENTEEISM

According to a web-based study, neck, back, or pelvic girdle pain and nausea and vomiting are pregnancy complications associated with medication use and sick leave in acute situations.^18,37^ Although not specific to workers in the hospital sector, the study covered pregnant workers and mothers of children under 1 year of age. In short, it addressed complications common in pregnancy^6,7,37^ that can be triggered or aggravated by work conditions.

Adverse gestational complications among health workers, such as miscarriage, abortion threat, premature labor, fetal anomalies, and intrauterine growth retardation,^15^ were also highlighted in other studies, although some are less specific.^6,7,37-39^ Bonzini et al. considered that the evidence on the risks of premature birth, low birth weight, and pre-eclampsia regarding the number of working hours and physical activities was insufficient to confirm the causal nexus between them. Nevertheless, they considered it would be prudent to advise against long hours of work, standing for a long time, and doing heavy physical work, especially late in pregnancy.^6^

Night shift work is a relevant occupational risk factor for pregnant women employed in hospitals. It increases the risk of miscarriage and getting sick the day after the work shift, and the number and duration of shifts longer than 12 hours are directly related to increased risks.^22-40^ In addition, the risk of hypertensive disorders of pregnancy (HDP) grows with the increase in consecutive shifts and quick returns after night shifts.^20,38^

The relationship between severe PPD and night work, on the other hand, seems to be more significant among pregnant workers in the hospital sector who stopped working the night shift after the first trimester of pregnancy, but not among those who continued working nights.^28^ The literature also points to associations between PPD and greater psychological demands, less time autonomy, and less perceived control over work and family.^41^

The prevalence of sick leave among working pregnant women tends to be higher in the first 32 weeks of pregnancy.^17^ The causes of long-term sick leave (> 20 days, continuous or intermittent) can be multiparity,^37^ low back pain before pregnancy, and illness or mental disorder,^27,42^ not necessarily related to working conditions.^17,26^

Short absences from work occasionally occur in the first 12 weeks due to episodes of temporary incapacity because of common contingency^24^ unrelated to work. From the third trimester onwards, occupational risk-benefits tend to be used by pregnant workers under high exposure to ergonomic, safety, hygiene, and psychosocial occupational risk factors.^26,42^

The studies analyzed do not support further conclusions about sick leave and absence due to occupational risk benefits because of different terminology, length of absence, workforce, and labor activity.

### WORK AND MATERNITY PROTECTION

There are several challenges to maternity and working life: lack of paid parental leave and associated financial penalties; loss of career opportunities; physical challenges related to pregnancy; decreased productivity; and the amount of time and effort involved in breastfeeding.^19^ Other factors such as interpersonal relationships and work adaptation, during pregnancy and on return to work to enable breastfeeding, are also challenges faced by workers.^25-44^

The existence of preventive or precautionary legislation for reassignment, especially if inserted in the health and safety legislation, favors pregnant workers continuing their work activities, with maintenance of their economic independence, and health protection.^16^ Furthermore, a literature review exploring pregnancy in the workplace recommends improving working conditions with social support and a proactive approach^45^ to sustain pregnant women’s capacity to work and ensure a healthy, risk-free work environment.^45,46^

The lack of consistent maternity protection policies, such as paid parental leave, can financially penalize families, reduce career opportunities and productivity, and make breastfeeding difficult.^19,47^ According to a multinational study, the importance of sick leave policies can be confirmed, considering that more than half of the population studied took leave during pregnancy. Women from countries with “low” sick leave policies were less likely to extend leave than women in countries with “medium” policies.^18^

Implementing legislation that protects maternity is subject to mechanisms that can hinder or facilitate application at the level of the individual, the physical and social environment, and the macro-social context. For example, unexpected adverse effects such as deteriorating employment relationships or discrimination can make it difficult to implement measures to protect maternity.^21,48,49^

The most significant barrier to the effectiveness of measures appears to be the delay between their conception and implementation. In many countries, preventive leave or sick leave is preferred at the expense of adaptations in the workplace or reassignment of workers to other sectors,^26,24,21^ showing inefficient implementation of maternity protection legislation or its inexistence.^49^

Furthermore, the conception and implementation of a maternity leave policy, as a protective measure, may be influenced by the logic that prevails in institutions such as family, state, profession, and the labor market.^43,49^ One example may be the difference in maternity policies between workers at large public hospitals and those in the private sector. The latter face a lack of social security, business strategies to escape maternity benefits, and limited options to extend leave.^30^

### STUDY LIMITATIONS

The heterogeneity of the articles did not allow collection of data to conduct a statistical meta-analysis. Therefore, the results are only descriptive.

The diversity of nomenclatures and meanings of some of the terms researched and the limited number of publications on the working conditions of pregnant workers in the hospital sector made searching for and selecting articles difficult. The results reported in the articles included in this review are very heterogeneous. They should be generalized with caution, observing sociocultural and economic differences in the labor market and organization of services.

## CONCLUSIONS

Few studies on working conditions and pregnancy focused on the hospital sector reinforce the need for specific studies. However, the review results support some inferences on pregnancy complications, associated occupational risk factors, the leading causes of absenteeism, maternity protection measures, and the challenges for pregnant workers’ careers and professional life in the hospital sector.

The most common complications in pregnancy for health professionals are pain in the neck, back, or pelvic girdle, nausea, and vomiting. The risk factors for adverse gestational complications such as miscarriage, abortion threat, premature labor, and HDP are night work and shift work, and risks increase with the number and length of shifts.

Due to differences in terminology, duration of absences, workforce, and work activity, it was not possible to draw precise conclusions about absences from work. However, the main reasons for absences are not work-related in early pregnancy.

Although adaptation of the work environment makes it possible to eliminate or minimize occupational risk factors, maintain work activity, and reduce illness and absence during pregnancy, putting pregnant women on leave from work still prevails. Task modification programs in the workplace for workers with partial and temporary incapacity for work are viable examples that can help pregnant women to continue working.

Motherhood imposes many challenges on workers due to inconsistent maternity protection legislation, lack of paid parental leave, financial losses, and career opportunities. In addition, interpersonal relationships and difficulties in adapting to work, during pregnancy or when returning from maternity leave, often add to the time and effort involved in breastfeeding.

It is necessary to consider mechanisms that may hinder or facilitate the application of maternity protection measures at different levels to reduce inequalities in access. Furthermore, it is essential to recognize discrimination against pregnancy and gender in the workplace and to insert specific maternity protection rules into OHS legislation at the national level.
